# Simulating Model Dielectric Functions of Dilute GaAs_1-x_N_x_ in the Far-Infrared to Ultraviolet Wavelength Regimes

**DOI:** 10.3390/ma19122575

**Published:** 2026-06-15

**Authors:** Devki N. Talwar, Hao-Hsiung Lin

**Affiliations:** 1 Department of Physics & Astronomy, University of North Florida, 1 UNF Drive, Jacksonville, FL 32224, USA; 2 Department of Physics, Indiana University of Pennsylvania, 975 Oakland Avenue, 56 Weyandt Hall, Indiana, PA 15705, USA; 3 Graduate Institute of Electronics Engineering and Department of Electrical Engineering, National Taiwan University, Taipei 106319, Taiwan; hhlin@ntu.edu.tw

**Keywords:** GaAs_1-x_N_x_/GaAs epilayers, dielectric functions, transfer matrix method, reflectivity/transmission spectra, spectroscopic ellipsometry, Green’s function, rigid-ion model

## Abstract

GaAs_1-x_N_x_/GaAs (001) (0 < x ≤ 0.037) tensile-strained epilayers are of considerable importance in optoelectronics due to their ability to offer large and resilient band structure engineering. Strain causes valence-band splitting, giant bandgap reduction and phonon frequency shifts. Optimum performance of III-V-Ns in long-wavelength lasers, infrared photodetectors, optical modulators, and multi-junction solar cells is contingent on their distinctive vibrational and optical characteristics. We report results of meticulous simulations of GaAs_1-x_N_x_ alloys to validate Fourier transform infrared (FTIR) reflectivity and spectroscopic ellipsometry (SE) data in the far-infrared and ultraviolet regions. The FTIR spectra showed strong reflectivity peaks and dips in the reststrahlen band region, linked to the transverse optical $\omega_{TO1}$ and longitudinal optical $\omega_{LO1}$ modes of the Ga-As bond and a high-frequency $\omega_{TO2}$ local vibrational mode of GaAs:N. Modified dielectric functions of GaAs_1-x_N_x_/GaAs epilayers are carefully evaluated using an improved Adachi’s semiemperical method to study the x and E-dependent optical constants. Focusing on the electronic band structures at critical points, this approach provided accurate analytical formulation to evaluate complex dielectric $\tilde{\varepsilon }$(E) and refractive indices $\tilde{n}$(E) for simulating reflectance spectra in a wide energy range with good agreement to the SE data.

## 1. Introduction

Mixing two different group III elements AIII and BIII in traditional III-V semiconductors while keeping the group-V element CV constant produces a cationic A_1-x_B_x_C ternary alloy [1,2,3,4,5,6,7,8,9,10]. The material typically forms a zinc-blende (zb) crystal structure of space group $F\bar{4}3m.$ Changing the molar fraction x allows the tuning of bandgaps $E_{g}\left(x\right)\;$ and lattice constants $a\left(x\right)$. For miscible solid solutions, the $a\left(x\right)$ follows a linear interpolation scheme and obeys Vegard’s rule [7,8]. Due to structural and chemical differences between atoms, the $E_{g}\left(x\right)$ has revealed a non-linear behavior with a small bowing coefficient [9]. Contrary to conventional III–V compounds, the incorporation of a small percentage of nitrogen (N) atoms x ~ 1–4% in GaAs (for instance) forms highly mismatched dilute GaAs_1-x_N_x_ alloys exhibiting a “giant” bandgap bowing [11,12,13,14,15,16,17,18,19,20]. This bowing cannot be described by a standard virtual crystal approximation [9] as it assumes a uniform potential and neglects local atomic fluctuations. Instead, this bowing is generally attributed to extreme structural and electronic perturbation as well as the mismatch between the electronegativity of N and As atoms. The phenomenon that relates to a significant reduction of $E_{g}\;$ is driven by the band anti-crossing model [21], where highly localized N-induced resonant states interact with the Γ-conduction band states of the GaAs matrix. This interaction triggers a strong repulsion that splits the conduction band into two sub-bands $E_{-}$ and $E_{+}$. Integration of In into dilute GaAs_1-x_N_x_ creates In_y_Ga_1-y_As_1-x_N_x_ quaternary alloys. Many researchers have independently tuned the lattice constant of In_y_Ga_1-y_As_1-x_N_x_ by adjusting x and y to match it with the technologically important GaAs and/or InP substrates [22]. In In_y_Ga_1-y_As_1-x_N_x_/GaAs (001) heterostructures [quantum wells/wires (QWs) [3,4] and superlattices (SLs) [9]], the incorporation of N introduces tensile strain, which compensates for compressive strain induced by In. By balancing opposing strains, the net lattice mismatch to GaAs or InP substrate is reduced, allowing for the growth of thicker, high-quality epitaxial layers without the formation of misfit dislocations.

Comparable strain-balancing principles have been investigated for mitigating stress-induced defects in other more heavily mismatched GaAs_1-x_N_x_ systems [11,12,13,14,15,16,17,18,19,20]. Their ultrathin epilayers are being used to achieve a wide range of bandgap energies, from ~1.45 eV to <0.8 eV. Moreover, III-V-Ns lack a center of symmetry while possessing strong non-linear optical properties to enable photonic applications for frequency generation and optical signal processing. These features are extensively employed in developing advanced and cost-effective, high-performance devices, including optical modulators (OMs), photodetectors (PDs), light-emitting diodes (LEDs), long-wavelength laser diodes (LW-LDs), vertical-cavity surface-emitting lasers (VCSELs), edge-emitting lasers, and high-efficiency multi-junction solar cells (MJ-SCs). Currently, both GaAs_1-x_N_x_ and In_y_Ga_1-y_As_1-x_N_x_ alloy epilayers are particularly sought after [11,12,13,14,15,16,17,18,19,20] for telecommunication applications, enabling 1.31 μm–1.55 μm emission and high-efficiency tandem solar cells.

Optical studies in dilute III-V-Ns have confirmed that increasing N leads to a dramatic non-linear redshift of the major bandgaps $E_{g}.$ To comprehend the electronic properties, several researchers have adopted a combination of experimental methods [22,23,24,25,26,27,28,29,30,31,32] focusing on their structural, optical, and transport characterization. The techniques that are commonly used include spectroscopic ellipsometry (SE) [26,27,28], photoluminescence (PL) [22,23,24,25], transmission/absorption, modulated reflectance (i.e., photoreflectance (PR)) [29,30,31,32], etc. Such methods are crucial and offer vital information regarding the complex nature of band structures $E_{g}\left(\vec{k}\right)$. By analyzing the second derivative of optical functions, it is possible to identify critical point (CP) energies in the Brillouin zone (BZ) where the probability of inter-band transition is high. In highly inhomogeneous GaAs_1-x_N_x_, no single method has adequately resolved the combined effects of large bandgap bowing and N-induced localization of energy states. SE is a powerful, non-invasive technique due to its high sensitivity in recognizing the subtle changes in the complex dielectric functions $\left(\tilde{\varepsilon }\right)$ and/or refractive indices $(\tilde{n}),$ making it essential for material characterization. Accurate knowledge of $\tilde{\varepsilon }\;or\;\tilde{n}$ for GaAs_1-x_N_x_ and In_y_Ga_1-y_As_1-x_N_x_ in the far-infrared (FIR) and ultraviolet (UV) regions is highly dependent on compositions x, y and photon energy E (≡ ħω). These optical functions are essential for designing photonic devices and quantifying how dilute III-V-N materials interact with light in resolving polarization, reflection, transmission and absorption behaviors across different electromagnetic energy regimes (EERs).

Earlier research on the optical properties of III-V-Ns was disproportionately focused on the near-infrared to visible energy range, ~0.8 μm–1.5 μm, due to their role in telecommunications, fiber optics and Si photonics. Limited studies exist, however, in the far-infrared (FIR) and ultraviolet (UV) regions. For In_y_Ga_1-y_As_1-x_N_x_ (y ≤ 0.33, x ≤ 0.007), the complex $\tilde{n}$ is measured using SE at photon energies below the fundamental bandgap [33]. For GaAs_1-x_N_x_, a significant rise in the optical absorption coefficient α [34] is linked to the decrease in $E_{g}$ with an increase in x. Based on SE findings, Grüning et al. [35] detected E_1_ CP energy. However, they were unable to resolve the x-dependent energy transitions. Šik et al. [36,37,38] earlier reported the optical properties of GaAsN/GaAs and GaAsN/InAs/GaAs SLs. In GaAs_1-x_N_x_, the effects of N and (001) biaxial tensile strain on the E_1_ and E_1_+Δ_1_ energy [39] were also studied. In the FIR region, phonon measurements by FTIR and Raman spectroscopy are crucial to understand lattice vibrations, yet they are less commonly applied to GaAsN/GaAs materials. While SE is a powerful tool to characterize optical properties of materials, its use in dilute III-V-Ns, focusing primarily on the UV region, is limited. This is due to the challenges of preparing high-quality, homogeneous alloys and by the localized nature of N-induced electronic states. Moreover, SE does not measure the optical constants (n, κ) directly. Mathematical modeling [40,41,42,43,44,45,46] and regression analysis are required for extracting them considering appropriate approximations.

This paper aims to report the results of methodical studies by carefully simulating the phonon and optical characteristics (cf. Section 2, Section 3 and Section 4) of GaAs_1-x_N_x_ alloys and epilayers. Limited SE measurements on dilute GaAs_1-x_N_x_ have motivated us to undertake this project for meticulously calculating the reflectivity [R(ω)] and transmission [T(ω)] spectra in the FIR (100 cm^−1^–600 cm^−1^) range (cf. Section 3.2) as well as studying the optical constants in the UV (≤6 eV) region (cf. Section 3.3). For bulk GaAs, we performed FIR reflectivity measurements using a Brüker IFS 120 v/S high-resolution Fourier transform infrared (FTIR) spectrometer. The exploitation of classical Lorentz–Drude oscillators is justified (cf. Section 3 and Section 4) in the framework of a transfer matrix method (TMM) [47] for evaluating the complex dielectric functions $\tilde{\varepsilon }\left(\omega \right)$ of both the epifilms and the substrate. For dilute GaAs_1-x_N_x_ alloys of different x ≤ 3.0%, we obtained the R(ω) and T(ω) spectra at near-normal ($\theta_{i}=0$) (cf. Section 3.2.1) and oblique $(\theta_{i}\neq 0$) incidence (cf. Section 3.2.2). A rigid-ion model (RIM) [48,49] in the Green’s function (GF) framework [50,51,52,53,54,55,56,57,58,59,60,61,62,63] is employed for comprehending the localized vibrational mode (LVM) of $N_{As}$ ($C_{As}$) in GaAs. A realistic bond orbital model (BOM) [64,65,66,67] is used for evaluating the lattice relaxation around N-impurities in GaAs. Due to differences in the size of N and As atoms, the force constant stiffening is accurately estimated from the change in bond lengths [53,54,55,56]. This is required in the GF methodology for defining the perturbation matrix $\overset{⃡}{P}(\omega )$ (cf. Section 4.2). The result of N (C)-isotopic shifts of LVM frequency compares favorably well with the FTIR and Raman scattering spectroscopy data [28,68,69,70,71,72,73]. Online digitization of limited SE results has permitted [41,42] a fast and efficient analysis of observed line shapes for assessing interband CP energies in the BZ. While a standard study is restricted to optically isotropic materials [41,42], its use in anisotropic systems has been demonstrated. In the UV region, we adopted a semiempirical approach for evaluating the dielectric functions $\tilde{\varepsilon }\left(E\right)$ and refractive indices $\tilde{n}\left(E\right)$ using the modified model dielectric functions (MDFs) [40,41,42,43,44,45,46]. For GaAs_1-x_N_x_, the values of MDFs are systematically obtained following Adachi’s approach [43,44,45,46] (cf. Section 3.3). Based on Kramers–Krönig (KK) transformation, the real ($\varepsilon_{1}$) and imaginary ($\varepsilon_{2}$) parts of $\tilde{\varepsilon }\left(E\right)$ are appropriately linked [43,44,45,46] to the electronic band structures $E_{j}\left(\vec{k}\right)$. Calculated results of LVMs using a lattice dynamical scheme in the GF method, as well as the $R\left(\omega \right),$ T(ω) spectra in the FIR and optical functions $\tilde{\varepsilon }\left(E\right)$ and $\tilde{n}\left(E\right)$ in the UV regions, are compared/contrasted against experimental/theoretical data (cf. Section 4.1, Section 4.2, Section 4.3 and Section 4.4). Concluding remarks are presented in Section 5.

## 2. Background

While the preparation of GaAs_1-x_N_x_ epilayers on GaAs has been successfully accomplished by molecular beam epitaxy (MBE) and/or metal–organic vapor phase epitaxy (MOVPE) techniques [22,23,24], investigations into their fundamental properties are still ongoing. Inquiries into their phonon and structural traits are either limited or sparse due to the significant lattice mismatch and low solubility of N. These factors have frequently resulted in the creation of nitrogen-related complex defect centers (viz., $N_{As}$-$N_{As}$, $N_{As}$-$\;N_{i}$) [28,68,69,70,71,72,73]. The high sensitivity of N to the local crystal field has triggered N-vacancy $V_{N}$, substitutional $N_{As}$ and interstitial $N_{i}$ defects in GaAs_1-x_N_x_, making it rather challenging to identify the exact point group symmetry of N-related centers, especially when multiple species are present.

### 2.1. Basic Properties of Dilute III-V-Ns

**Figure 1:**
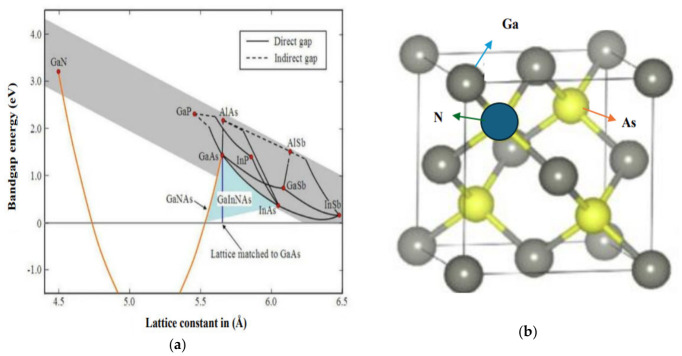
(**a**) Bandgap (eV) vs. lattice constant (Å) for III-V alloys, showing lines of lattice match to GaAs for GaInNAs in the region applicable to long-wavelength fiber systems: 1.3 µm–1.55 µm (0.954 eV–0.8 eV) [24,25,26,27,28,29,30,31,32,33,34,35]. (**b**) Crystal structure of N (blue circle) occupying an As (green circle) site in zb GaAs material [68,69,70,71,72,73].

Addition of N to GaAs and/or to InGaAs acts as a highly effective way of dramatically decreasing the bandgap energy $E_{g}$ (see Figure 1a). This outcome has often been referred to as a giant bowing effect in the band structure of dilute III-V-N alloys. The huge reduction in $E_{g}$ is attributed to a higher electronegativity of N compared to As atoms, which strongly perturbs the host lattice. Adding N to InGaAs allows the tuning of $E_{g}$ to even smaller values [24,25,26,27,28,29,30,31,32,33,34,35]. This has made InGaAsN alloys suitable for designing optoelectronic devices including lasers, PDs and MJ-SCs, to operate at longer wavelengths for fiber optic communications, while maintaining lattice-matched, strain-free conditions on GaAs. The use of active InGaAsN/GaAs QWs to fabricate VCSELs in the 1.31 μm–1.55 μm (0.95 eV–0.80 eV) range represents a significant achievement by leveraging them with a mature AlAs/GaAs distributed Bragg reflector mirror technology [24,25,26,27,28,29,30,31,32,33,34,35].

Nitrogen substituting for As in GaAs $\left(N_{As}\right)$ is a classic and most significant example of an isoelectronic impurity (see Figure 1b). Since N lacks p orbitals in its core states, it acts as a much stronger electron trap due to a higher electronegativity and small atomic radius than the As atom. Moreover, the difference in Ga-As and Ga-N bond lengths leads to lattice relaxation and an important local redistribution of charge density. These combined effects result in a perturbed charge distribution around the N atom, creating a region of lower potential energy that is appealing to electrons. Unlike charged impurities (cf. Section 3) that interact via long-range Coulomb potentials, $N_{As}$ acts as a neutral impurity. It has a short-range potential, meaning that it only affects the core region of the defect site rather than the distant lattice structure [68,69,70,71,72,73].

### 2.2. Structural and Phonon Characteristics

In GaAs_1-x_N_x_, the high-resolution x-ray diffraction (HR-XRD), atomic force microscopy (AFM) and transmission electron microscopy (TEM) are frequently used to analyze the structural and chemical distribution of atoms. Such high spatial resolution methods have provided insights into the crystalline quality of materials with N incorporation by focusing on lattice strain and surface morphology. The evaluation of N composition x in GaAs_1-x_N_x_ is achieved by electron probe microanalysis (EPMA) in conjunction with energy-dispersive spectroscopy (EDS) or wavelength-dispersive spectrometry (WDS) [22,23,24,25,26,27,28,29,30,31,32]. Hall measurements are widely employed with a van der Pauw technique for characterizing the electrical properties of dilute GaAsN and other III-V-Ns. This approach has been particularly important for analyzing metastable, low-temperature-grown materials to comprehend the impact of defects and annealing on the performance of electronic devices. While the optical traits of GaAsN-based alloys are commonly examined by PL to study the variation of $E_{g}$, the identification of their phonon characteristics by FTIR is, however, often limited. SE measurement is normally exploited in alloyed epifilms for determining the composition gradient and their microstructures. This method has also been employed in samples to distinguish between separate phases (i.e., crystalline, amorphous, void) in determining optical constants and understanding their band structures [22,23,24,25,26,27,28,29,30,31,32].

The LVMs of N in GaAs_1-x_N_x_ have been analyzed [28,68,69,70,71,72,73,74,75,76,77,78] by combining high-resolution FTIR or Raman spectroscopy with atomistic theoretical modeling. While spectroscopy has identified the specific vibrational frequencies corresponding to isolated substitutional nitrogen $N_{As}$ and N clusters, the modeling has validated these structures by calculating their LVMs. In different semiconductor materials, Raman spectroscopy measurements in the backscattering geometry have often utilized Ar-ion lasers for investigating impurity or defect-related, high-frequency, non-lattice vibrations. In III-V-Ns, rapid thermal annealing has frequently been used to improve the material’s quality and to reduce the concentration of certain types of N complex defects. Earlier, several IR studies identified specific N pair centers, which appeared as sub-peaks near the main $N_{As}$ absorption band [28,68,69,70,71,72,73,74,75,76,77,78].

## 3. Theoretical Section

While research on the growth of binary zb GaAs and GaN materials has progressed significantly [22,23,24], limited experimental and theoretical efforts exist for comprehending the structural and vibrational behavior of ternary GaAs_1-x_N_x_ and quaternary alloys [74,75]. Careful evaluation of the lattice dynamical aspects of GaAsN alloyed thin films leads to considerable phonon-assisted optical absorption processes that impact on the optimization of device structures for achieving high-performance optoelectronic units.

### 3.1. Lattice Dynamics

Lattice dynamics of GaAs and GaN materials are fundamental for understanding the vibrational properties and structural stability of ternary GaAs_1-x_N_x_ alloys. Phonon dispersions $\omega_{j}\left(\vec{q}\right)$ of perfect crystals are extensively studied by experimental and theoretical methods [48,49,76,77,78,79,80]. Inelastic neutron scattering (INS) spectroscopy [78] is considered one of the most accurate methods for measuring $\omega_{j}\left(\vec{q}\right)$. Experimental data obtained through INS and RSS have served as a diagnostic benchmark for developing, validating and refining different theoretical methods [48,49,76,77,78].

For perfect GaX (X = As, N), ab initio studies are employed [76,77,78] to simulate $\omega_{j}\left(\vec{q}\right)$ and other phonon characteristics without considering experimental data or empirical parameters. First-principles calculations have commonly employed either ABINIT software or the Quantum-Espresso programs [76,77,78]. A realistic RIM [48] is adopted here to obtain the inter-atomic force constants (IFCs) of GaAs and GaN [48,49] (see Table 1) to study their $\omega_{j}\left(\vec{q}\right)$ and one-phonon density of states,  g(ω). Using least-square fitting procedures [49], the method of calculating IFCs is briefly described in the Section Rigid-Ion Model. In the framework of a RIM, we also employed the Green’s function (GF) theory, which helped predict the x-dependent $\omega_{j}\left(\vec{q}\right)\;and$ $g\left(\omega \right)$ for GaAs_1-x_N_x_ alloys [79,80].

#### Rigid-Ion Model

To understand the phonon characteristics of perfect GaX (X = As, N) materials, one requires a realistic lattice dynamical scheme. Here, we have adopted a RIM developed by Kunc [48] for zb materials. The model considers both short-range interactions up to 2nd NNs (A, B, $C_{\kappa }$, $D_{\kappa }$, $E_{\kappa }$, $F_{\kappa }$ with κ = 1,2) and a long-range Coulomb interaction Z_eff_ [48,49].

Atoms in GaX are identified using indices *l* and κ. The term *l* represents the number of unit cells, while κ signifies the two types of atoms (i.e., κ = 1 (Ga) and κ = 2 (X)). Assuming that the Ga atom (κ = 1) is located at the origin (0, 0, 0) and the X atom is along the body diagonal of the zb lattice (κ = 2) with its location at x = y = z = *a*_0_/2 [48], the unit cell volume attains the value $2a_{0}^{3}.$ The short-range coupling matrices between the NN Ga-X atoms are defined as [48,49]

(1) $$\left(\begin{array}{ccc} A & B & B\\ B & A & B\\ B & B & A \end{array}\right),$$

and the interactions between the 2nd NN Ga-Ga and X-X atoms as [48]

(2) $$\left(\begin{array}{ccc} C_{1} & D_{1} & E_{1}\\ D_{1} & C_{1} & E_{1}\\ -E_{1} & -E_{1} & F_{1} \end{array}\right)\;\mathrm{and}\;\left(\begin{array}{ccc} C_{2} & D_{2} & {-E}_{2}\\ D_{2} & C_{2} & {-E}_{2}\\ E_{2} & E_{2} & F_{2} \end{array}\right),$$

In RIM, the atomic displacements $u_{\alpha }$ of point ions from their equilibrium positions are assumed to be rigid and non-polarizable. In this formalism, the atomic displacements of the jth vibrational mode are written in terms of plane waves with wave vector $\vec{q}$ and $\omega_{j}(\vec{q})$ as [48]

(3) $$u_{\alpha }\left(l\kappa |\vec{q}j\right)=\frac{1}{\sqrt{M_{k}}}e_{\alpha }\left(\kappa |\vec{q}j\right)exp(i(\vec{q}.\vec{x}\left(l\kappa \right)-\omega_{j}(\vec{q})t)),\;\mathrm{with}\alpha =x,y,\;\mathrm{and}\;z$$

In Equation (3), the term t identifies time, $\vec{x}\left(l\kappa \right)$ and $M_{\kappa }$ represent, respectively, the position and mass of the $\left(l\kappa \right)$ atom. With harmonic approximation for the potential energy, one can write the equations of motion in the RIM as [48]

(4) $$\omega_{j}^{2}\left(\vec{q}\right)e_{\alpha }\left(\kappa |\vec{q}j\right)=\sum_{k^{\prime }\beta }D_{\alpha \beta }^{sC}(\kappa \kappa^{\prime }|\vec{q})e_{\beta }\left(\kappa^{\prime }|\vec{q}j\right);\kappa {,\kappa }^{\prime }=1,2$$

where $D_{\alpha \beta }^{sC}\left(\kappa \kappa^{\prime }|\vec{q}\right)[\equiv D_{\alpha \beta }^{s}\left(\kappa \kappa^{\prime }|\vec{q}\right)+D_{\alpha \beta }^{C}\left(\kappa \kappa^{\prime }|\vec{q}\right)]$ represents the dynamical matrix comprising both the short $D_{\alpha \beta }^{s}\left(\kappa \kappa^{\prime }|\vec{q}\right)$ and long-range $Coulomb\;D_{\alpha \beta }^{C}\left(\kappa \kappa^{\prime }|\vec{q}\right)$ interactions.

One must note that the components of eigen vectors for each frequency $\omega_{j}\left(\vec{q}\right)$ satisfy the familiar orthogonality, i.e.,

(5) $$\sum_{\alpha k}e_{\alpha }^{*}\left(\kappa |\vec{q}j\right)e_{\alpha }\left(\kappa |\vec{q}j^{\prime }\right)=\delta_{jj^{\prime }}$$

and closure relations

(6) $$\sum_{j}e_{\alpha }^{*}\left(\kappa^{\prime }|\vec{q}j\right)e_{\beta }\left(\kappa |\vec{q}j^{\prime }\right)=\delta_{\kappa \kappa^{\prime }}\delta_{\alpha \beta }.$$

For GaX, the IFCs (A, B, $C_{\kappa }$, $D_{\kappa }$, $E_{\kappa }$, $F_{\kappa }$ and Zeff) of RIM are carefully evaluated (see Table 1) using least-square fitting procedures described elsewhere [49]. These parameters are related to the elastic constants $c_{ij}$, lattice constants $a_{0}$ as well as the phonon frequencies at Γ, X, and L critical points (CPs) of perfect materials [49]. The model parameters have resulted in a reasonably good fit—posteriori to the INS, RSS and/or ab initio results [76,77,78] of phonon dispersion $\omega_{j}\left(\vec{q}\right)$ along high-symmetry directions (Γ→X→K→Γ→L→X→W→L) in the BZ (cf. Section 4.1). However, the comparison of RIM phonon values is less satisfactory at certain CPs near the edge of the BZ (Table 2).

We must state that the RIM adopted here represents ions in a lattice as unyielding, point-like charged particles interacting via the short-range IFCs and long-range Coulomb interactions. In this framework, an applied strain can simply translate the sub-lattices rigidly. As the model only accounts for point charges, it limits the calculated polarization to the product of macroscopic strain and Born’s effective charge [49]. While the RIM is widely used to study phonon dispersions $\omega_{j}\left(\vec{q}\right)$, it suffers from several key physical limitations: (a) Due to the lack of dynamic electron polarizability, the model underestimates splitting between $\omega_{LO(\Gamma )}-\omega_{TO(\Gamma )}$ modes, and (b) as the zb structure lacks inversion symmetry, it provides piezoelectric coefficients that fail to capture the deformation dipoles when the relative sublattices shift [48].

### 3.2. Analyses of FTIR Spectra

Using Snell’s law and Maxwell’s equations, a TMM has been developed [47] to study the $R\left(\omega \right)\;$ and $T\left(\omega \right)$ spectra of a multi-layer structure grown on a substrate in the FIR region. The structural make-up is considered by stacking j-number of ultrathin films, each with thickness d_f_ and refractive index ${\tilde{n}}_{f}(\omega )(=\sqrt{{\tilde{\varepsilon }}_{f}\left(\omega \right)})$. Both d_f_ and ${\tilde{n}}_{f}(\omega )$ are required (cf. Section 3.2.1) for modeling the $R\left(\omega \right)$ and $T\left(\omega \right)\;$ spectra of single GaAs_1-x_N_x_ layers of different x grown on a GaAs (001) substrate at near-normal $(\theta_{i}=0)\;$(cf. Section 3.2.1) [61,62] and oblique $(\theta_{i}\neq 0)\;$ incidence (cf. Section 3.2.2) [81].

#### 3.2.1. Dielectric Response Function at $\theta_{i}=0$

At near-normal ${(\theta }_{i}=0)\;and\;oblique\;{(\theta }_{i}\neq 0\;)\;incidence$, the dielectric response function of a polar film ${\tilde{\varepsilon }}_{f}(\omega ,\vec{q})$ (or substrate $\;{\tilde{\varepsilon }}_{s}(\omega ,\vec{q})$) has been frequently assessed by adopting (a) the Lorentz method of optical lattice phonons ${\tilde{\varepsilon }}_{l}(\omega ,\vec{q})$ and (b) the Drude model of free charge carriers ${\tilde{\varepsilon }}_{e}(\omega ,\vec{q})$. In the limiting case with wavevector $\vec{q}$ ⟶ 0, the general form of the Lorentz–Drude approach for computing ${\tilde{\varepsilon }}_{f}(\omega ,\vec{q})$ for GaAs_1-x_N_x_ film takes the form [61,62]

(7) $${\tilde{\varepsilon }}_{f}(\omega )={\tilde{\varepsilon }}_{l}\left(\omega \right)+{\tilde{\varepsilon }}_{e}(\omega ),$$

with

(8) $${\tilde{\varepsilon }}_{f}\left(\omega \right)=\left[\varepsilon_{\infty }+\sum_{j=1-2}\frac{S_{j}\omega_{TOj}^{2}}{\omega_{TOj}^{2}-\omega^{2}-i\Gamma_{j}\omega }\right]-\frac{\omega_{p}^{2}}{\omega (\omega +i\gamma_{p})}$$

The term $\omega_{p}=\sqrt{\frac{4\pi \eta e^{2}}{{m_{e}}^{*}\varepsilon_{\infty }}}$ in Equation (8) represents the plasma frequency; η stands for free charge carrier density; $m_{e}^{*}\;$ is the effective electron mass; e is the electron charge; $\gamma_{p}$($\Gamma_{j}$) signifies plasmon (phonon) damping coefficient; $\omega_{TOj}$ symbolizes the TO phonon frequency; $S_{j}=\varepsilon_{\infty }\left[\frac{\omega_{LOj}^{2}-\omega_{TOj}^{2}}{\omega_{TOj}^{2}}\right]$ is the oscillator strength; ω stands for the frequency of incident light; and $\varepsilon_{\infty }$ is the high-frequency dielectric constant. The sum over j = 1–2 covers the appropriate Lorentz oscillators of GaAs and GaN binary materials involved in the ternary GaAs_1-x_N_x_ alloys.

The dielectric function of the substrate ${\tilde{\varepsilon }}_{s}(\omega )\;\left(\equiv GaAs\right)$ is calculated by using [61,62]

(9) $${\tilde{\varepsilon }}_{s}(\omega )=\frac{S\omega_{TO}^{2}}{\omega_{TO}^{2}-\omega^{2}-i\omega \Gamma }-\frac{\omega_{p}^{2}}{\omega (\omega +i\gamma_{p})}$$

The dielectric function of the film ${\tilde{\varepsilon }}_{f}\left(\omega \right)$(or substrate ${\tilde{\varepsilon }}_{s}\left(\omega \right)$) is related to the complex refractive index by ${\tilde{n}}_{f}=\sqrt{{\tilde{\varepsilon }}_{f}}$ (or ${\tilde{n}}_{s}=\sqrt{{\tilde{\varepsilon }}_{s}}).$ The best-fit Lorentz–Drude parameter values used to simulate R(ω) and T(ω) for GaAs_1-x_N_x_ and GaAs are reported in Table 3.

#### 3.2.2. Dielectric Response Function at $\theta_{i}\neq 0$

In polar films of thickness d_f_ (with d_f_ << λ), the oblique incidence $\theta_{i}\neq 0$ of FIR radiation breaks the symmetry and allows the light to couple with both the $\omega_{TO}$ and $\omega_{LO}$ phonons [81]. As the electric field vector perpendicular to the plane of incidence (i.e., parallel to the film surface (s-polarization)) cannot penetrate the film normal to its surface, the transmission T_s_ can only excite the $\omega_{TO}$ mode where the atomic motions are strictly parallel to the film. The electric vector lying in the plane of incidence generates an electric field component normal to the film boundary (p-polarization). This normal field induces surface charges that allow the radiation to couple to $\omega_{LO}$ modes where the atomic vibrations are perpendicular to the film surface. Consequently, in the T_p_ transmission, one observes minima at both $\omega_{TO}$ and $\omega_{LO}$. For a deeper dive into the original theoretical framework of this phenomenon, we refer to the original paper [81]. Earlier, we successfully analyzed the experimental R(ω) and T(ω) spectra at $\theta_{i}=0$ and $\theta_{i}\neq 0$ for ternary alloy Cd_x_Zn_1-x_Te (0 < x < 1) and CdTe_1-x_Se_x_ (0 < x ≤ 0.35) epifilms [82,83]—corroborating Berreman’s effect [81]. This methodology is applied to GaAs_1-x_N_x_ (cf. Section 4.3).

### 3.3. Analyzing Spectroscopic Ellipsometry Data

To extract the optical constants from SE data in the UV region of layered structured materials, parametric MDF methods are frequently employed to ensure KK consistency [40,41,42,43,44,45,46]. In the visible range, the Cauchy model (often with an Urbach tail) [84] fits transparent dielectrics, while the Zollner method [85] is exploited to precisely characterize native oxides on different materials. These methods have provided reasonably accurate and efficient dielectric functions $\tilde{\varepsilon }$(E) [≡ $\varepsilon_{1}\left(E\right)+i\;\varepsilon_{2}\left(E\right)]$ of thin films. For compound semiconductors, the use of general parametric models is highly effective for fitting SE data to describe CPs and line shapes, accounting for complex band structures [40,41,42]. Based on different approaches, the above methods can be divided into three categories [85] depending on how they incorporate the CP energies and broadenings to describe the complex dielectric functions $\tilde{\varepsilon }$(E) [40,41,42].

An extension to Adachi’s method suggested by Rakić and Majewski [46] is applied here. This modification [46] involved augmenting the original model [40,41,42] by introducing a Gaussian-like broadening function instead of the standard Lorentzian-type function in deriving final expressions for the dielectric constants. To comprehend Adachi’s foundational approach, researchers are typically referred to the work outlined in Refs. [43,44,45]. To simulate $\tilde{\varepsilon }$(E) (≡$\;\varepsilon_{1}(E)+i\;\varepsilon_{2}(E)),$ we have carefully included the contributions of inter-band electronic transitions at CPs (e.g., i.e., $E_{0}$, $E_{0}+\Delta_{0}$, $E_{1},\;and\;E_{1}+{∆}_{1}$, etc.) within the framework of joint density of states [46]. These values are usually initialized using established theoretical band structure models, perturbation theory and/or pseudopotential methods to match the exact resonance locations [40,41,42]. Mathematical equations for $\tilde{\varepsilon }$(E) described in the Section Modified Adachi’s Model Dielectric Function serve as a simulator, where energy E is an independent variable. This approach (cf. the Section Modified Adachi’s Model Dielectric Function) relies on the values of CPs as well as amplitudes $A_{k}$ of transition energies $E_{k}$, $Gaussian\;broadening\;\Gamma_{k}$ and distribution parameters $\alpha_{k}$, among others. The model parameters are empirically extracted via non-linear regression analysis (such as the Levenberg–Marquardt algorithm [75]) by fitting the calculated dielectric functions to the experimental SE data [40,41,42].

#### Modified Adachi’s Model Dielectric Function

Based on one-electron inter-band transition, Adachi’s method [40,41,42] relies on the parabolic band approximation while assuming energy-independent momentum-matrix elements. A detailed introduction to Adachi’s semi-empirical approach is described elsewhere [43,44,45]. For evaluating the contributions from different CPs, except for $E_{0}$ and $E_{0}+\Delta_{0}$ transitions to dielectric function $\tilde{\varepsilon }\left(E\right),$ we have followed Rakić and Majewski [46] and replaced the Lorentzian broadening functions with Gaussian-like functions. Next, we describe the contributions of CPs to $\tilde{\varepsilon }(E)$ in the BZ with appropriate expressions for assessing MDFs, particularly where the joint density of states exhibits singularities.

The $E_{0}$, and $E_{0}+\Delta_{0}$ transitions in zb materials occur at the center of the BZ (3D $M_{0}).$ Assuming that the bands are parabolic and using KK relations, one can obtain the contribution of these energy transitions to $\tilde{\varepsilon }(E)$ by using [43,44,45]

(10) $${\tilde{\varepsilon }}_{j}\left(E\right)=A_{j}E_{j}^{-3/2}\left\{c_{j}^{-2}[2-(1+c_{j}{)}^{0.5}-(1-c_{j}{)}^{0.5}]\right\},$$

with $c_{j}=\frac{\left(\hbar \omega +i\Gamma_{j}\right)}{E_{j}}$ [j = 0 and 1 for $E_{0}$, and $E_{0}+\Delta_{0}$, respectively]. The terms $A_{j}$, $E_{j}$, and $\Gamma_{j}$ are respectively the amplitude, transition energy, and broadening parameter of each CP structure.

The $E_{1}\;{and\;E}_{1}+{∆}_{1}$ transitions are of 3D $M_{1}$ type that take place along the <111> directions at Λ or L points in the BZ. As the longitudinal effective mass of $M_{1}$ is much larger than the transverse effective mass, such energy transitions are usually treated as 2D $M_{0}$ CPs [43,44,45]. The contribution of $E_{1}$ and $E_{1}+{∆}_{1}$ CP structures to $\tilde{\varepsilon }(E)$ is given by:

(11) $${\tilde{\varepsilon }}_{k}\left(E\right)={-A}_{k}c_{k}^{-2}\ln\left(1-c_{k}^{-2}\right),$$

with $c_{k}=\frac{\left\{\hbar \omega +i\Gamma_{k}\exp(-\alpha_{k}\left[E_{k}-\hbar \omega {]}^{2}\right)\right\}}{E_{k}}$ [k = 1 and 2 for $E_{1}$, and $E_{1}+{∆}_{1}$, respectively]. The terms $A_{k}$, $E_{k}$, $\Gamma_{k}$ and $\alpha_{k}$ are respectively the amplitude, transition energy, broadening and distribution parameter of energy-dependent broadening of the $E_{1}$ and $E_{1}+{∆}_{1}$ CP structures.

The contributions to $\tilde{\varepsilon }\left(E\right)$ due to the ground-state Wannier-type 2D excitons are considered at the $E_{1}$, and $E_{1}+{∆}_{1}$ CP structures. Exciton-induced dielectric susceptibility is approximated using a single damped Lorentzian (DL) line shape with an energy-dependent broadening term [43,44,45,46]:

(12) $${\tilde{\varepsilon }}_{kx}\left(E\right)=\frac{A_{kx}}{E_{k}-\hbar \omega -i\Gamma_{kx}\exp(-\alpha_{kx}\left[E_{k}-\hbar \omega {]}^{2}\right)},$$

where k = 1 and 2 for $E_{1}$ and $E_{1}+{∆}_{1}$, respectively. The terms $A_{kx}$, $E_{k}$, $\Gamma_{kx}$ and $\alpha_{kx}$ are the amplitude, transition energy, broadening and distribution of energy-dependent broadening of excitons.

Contributions to $\tilde{\varepsilon }\left(E\right)$ due to $E_{0}^{\prime }$ and $E_{2}$ CP structures are characterized by damped harmonic oscillators [43,44,45,46]:

(13) $${\tilde{\varepsilon }}_{m}\left(E\right)=\frac{A_{m}}{{1-(E/E}_{m}{)}^{2}-i{\left(E/E_{m}\right)\Gamma }_{m}\exp(-\alpha_{m}\left[{E-E}_{m}{]}^{2}\right)},$$

with energy-dependent broadening terms [m = 2 and 3 for $E_{0}^{\prime }$ and $E_{2}$, respectively]. The quantities $A_{m}$, $E_{m}$, $\Gamma_{m}$ and $\alpha_{m}$ are the amplitude, transition energy, broadening and distribution parameter.

Combining all these impacts and using Equations (10)–(13), the complex dielectric function $\tilde{\varepsilon }(E)$ can be expressed as the sum of terms [43,44,45]:

(14) $$\tilde{\varepsilon }(E)=\;{\tilde{\varepsilon }}_{j}\left(E\right)+{\tilde{\varepsilon }}_{k}\left(E\right)+{\tilde{\varepsilon }}_{kx}\left(E\right)+{\tilde{\varepsilon }}_{m}\left(E\right)+\varepsilon_{\infty }$$

## 4. Numerical Computations: Results and Discussion

Lattice dynamics of binary GaAs and GaN materials is critical for understanding the vibrational properties of dilute ternary GaAs_1-x_N_x_ alloys. Phonon dispersions $\omega_{j}\left(\vec{q}\right)$ of the perfect zb crystals are extensively studied by experimental and theoretical methods [48,49,76,77,78]. Inelastic neutron scattering [78] spectroscopy is viewed as a premier and high-precision technique for measuring $\omega_{j}\left(\vec{q}\right)$. Experimental results obtained through INS and RSS have served as a diagnostic benchmark [48,49,76,77,78] for developing, validating and refining different theoretical methods.

### 4.1. Phonon Dispersions

**Figure 2:**
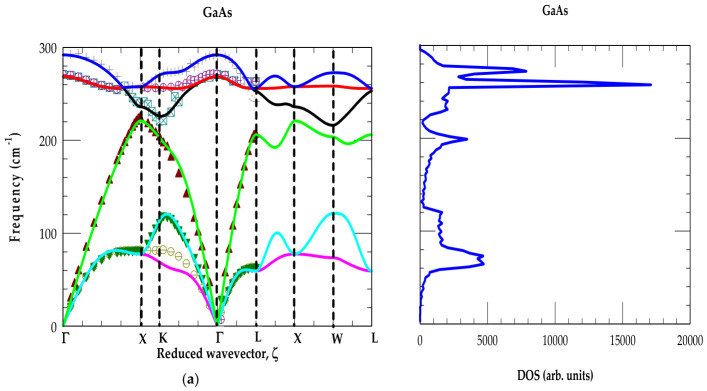

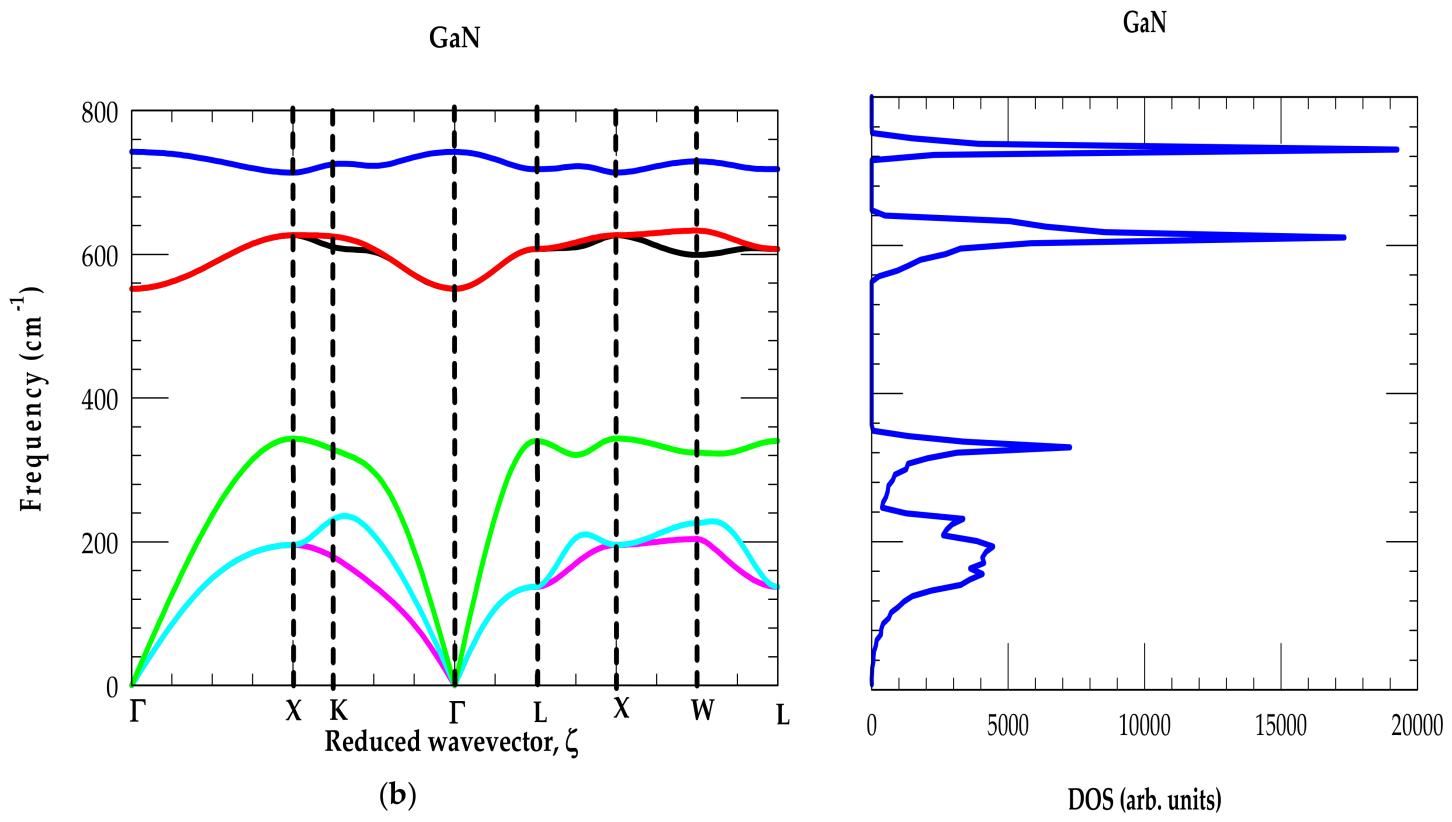
Comparative study of RIM $\omega_{j}\left(\vec{q}\right)$ (left panel) and density of states g(ω) (right panel) for: (**a**) GaAs, and (**b**) GaN. Full colored lines indicate our results, while different symbols represent data from INS [78] and/or ab initio calculations [76,77].

We have adopted a realistic RIM to obtain the IFCs (see Table 1) for GaAs and GaN materials [48,49] to study their $\omega_{j}\left(\vec{q}\right)$ and one-phonon density of states, g(ω). Results (displayed in Figure 2a,b) are compared with the existing INS [78], RSS, and ab initio methods [76,77]. Consistent with first-principles calculations [76,77] and experimental [78] data, our RIM report has shown identical trends (see Table 2) for $\omega_{j}\left(\vec{q}\right)$ and $g\left(\omega \right).$ Due to the variation of anion As and N masses, the study revealed significant differences in phonon frequencies. The increasing mass ratio of the two atoms has clearly demonstrated the key features across the GaN⟶ GaAs series: (a) in GaN, the highest frequencies of longitudinal optical LO ($\omega_{LO}$) and transverse optical TO ($\omega_{TO}$) modes are seen with a wider separation between its acoustic and optical branches, and (b) in GaAs, the LO,TO phonons are lower than GaN due to the heavier mass of the As atom.

In GaAs, a negligible or non-existent phonon gap exists between its acoustic and optic branches (cf. Figure 2a). The main reason for such an insignificant phonon gap in phonon dispersions of GaAs is the close proximity of Ga and As atomic masses. We have incorporated the RIM with GF methodology that helped us predict the x-dependent $\omega_{j}\left(\vec{q}\right)\;\;and$ $g\left(\omega \right)\;$[79] for GaAs_1-x_N_x_ alloys.

### 4.2. Local Vibrational Modes

In GaAs, the simplest isolated defect occupying the host As (κ = 2) lattice atom is a light substitutional N-impurity (see Figure 1b). This iso-electronic $N_{As}$ impurity of $T_{d}\;$ symmetry creates a high-frequency LVM (cf. the Section Rigid-Ion Model). In the RIM framework using GF theory, the perturbation matrix $\overset{⃡}{P}(\omega )$ can be defined to include both the variation of atomic mass at the impurity site and an NN force constant, u. Appropriate changes are expressed by [57]:

(15) $$\varepsilon_{2}=\;(M_{2}-M_{2}^{imp})/M_{2},$$

(16) $$u=(A-A^{\prime \prime })/A=(B-B^{\prime \prime })/B=1-b,$$

with an impurity mass $M_{2}^{imp}$ occupying site κ = 2. Following Vandevyver and Plumelle [57], we considered the impurity-host interaction by using a single dimensionless parameter, u. In Equation (16), the stipulation of bA = bB for delineating $\overset{⃡}{P}(\omega )$ hardly affects the high-frequency LVM. However, imposing this condition satisfies the rotational invariance requirement, which is explicitly invariant with respect to translations and crystal-symmetry operations [57].

#### 4.2.1. Perturbation Matrix

In any study of impurity vibrations, the most important problem has been to give an adequate representation of $\overset{⃡}{P}(\omega )$. This perturbation matrix must include the effects of defects on the short- and long-range Coulomb interactions, lattice relaxation and charge state splitting, etc. To the best of our knowledge, no unified theory exists where all these factors are properly included. In the framework of RIM using GF theory, one can avoid some of these effects to construct $\overset{⃡}{P}\left(\omega \right)$ by using the scaling properties and chemical trends in the short-range interactions of the host crystal’s dynamical matrix [57].

#### 4.2.2. Local Distortions of Isolated Defects

Computationally efficient first principles BOM has been successfully used [64,65,66,67] for calculating impurity-induced lattice distortions in semiconductors. The method creates a robust mathematical basis by employing orthogonal and normalized sp^3^ hybrids to describe tetrahedral coordination. The BOM describes the defect environment by balancing attractive covalent bond energy (arising from orbital overlap) with repulsive energy (originating from core orthogonality). For estimating local lattice relaxations around isolated defects, the model requires minimizing the total bond energy $E_{b}$ for stable atomic geometries to resolve how NN atoms adjust to structural configurations. Obviously, $E_{b}$ dictates how much surrounding atoms of a substitutional defect relax inward or outward. This continuous relaxation process directly balances both the radial attraction/repulsion of bond-stretching and the angular resistance of bond-bending. By treating the defect environment as a sum of hybrid covalent, overlap and repulsive energies, the model accurately predicts the alteration of surrounding bond lengths caused by isolated defects [64,65,66,67].

Replacing an impurity atom with the host lattice atom creates a new bond-orbital interaction with its NNs. Distortion around the impurity atom can cause changes in the bond energies between the 1st NN impurity-host $∆E_{b}^{1}$ and 2nd NN host-host atoms $∆E_{b}^{2}$. If a substituted impurity forms a bond with the host lattice atoms having higher average hybrid energy than the host-host bonds, NN impurity-host atoms undergo inward relaxation. This causes stiffening in the impurity-host bonding. Conversely, if the impurity bond results in a lower average hybrid energy, it induces an outward distortion, causing softening in the impurity-host interaction [64,65,66,67].

**Figure 3:**
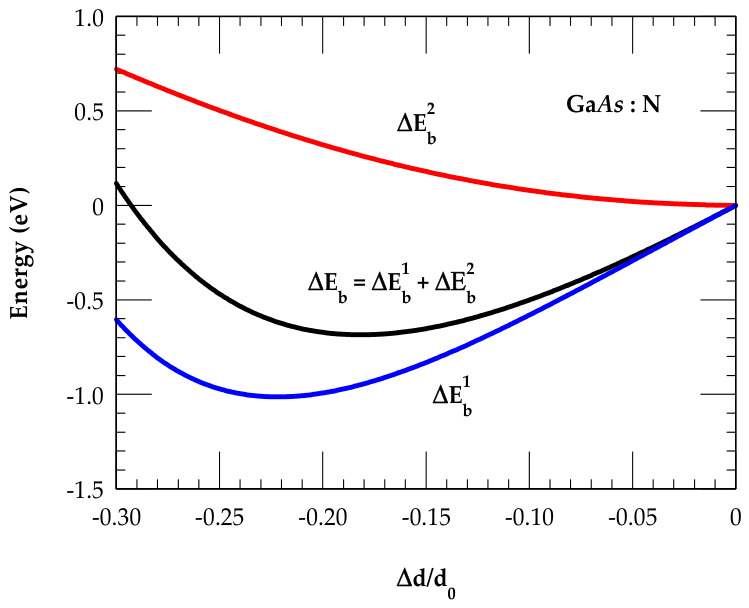
The variation of change in impurity–host $∆E_{b}^{1}$ (blue color), host–host $∆E_{b}^{2}$ (red color) and total $∆E_{b}$ ($\equiv ∆E_{b}^{1}+∆E_{b}^{2}$) (black color) bond energies [64,65,66,67] versus $∆d/d_{0}$ for GaAs:N (see text).

Earlier, we accurately estimated the local distortions $∆d/d_{0}$ by minimizing the total change in bond energy $∆E_{b}$, i.e., $\partial ∆E_{b}/\partial ∆d=0\;$, in zb semiconductors due to several isolated substitutional impurities occupying either cation or anion sites [65,66,67]. For GaAs:N, the calculated variations of impurity–host $∆E_{b}^{1}$, host–host $∆E_{b}^{2}$, and total $∆E_{b}$ ($\equiv ∆E_{b}^{1}+∆E_{b}^{2}$) change in the bond energies [64,65,66,67] are displayed in Figure 3 as a function of $∆d/d_{0}.$ Here, $d_{0}$ is the bond length of the perfect GaAs.

For isolated $N_{As}$ in GaAs, we estimated $∆d/d_{0}$ [= − 0.18] from the minimum of the total change in bond energy (see Figure 3). In the GF theory, this value is used for calculating the stiffening of force constant variation u (cf. Section 4.2.3) in the perturbation matrix $\overset{⃡}{P}(\omega )$ to study the LVM of N_As_ in GaAs.

#### 4.2.3. Impurity Modes

Earlier, we evaluated the Green’s function matrix ${\overset{⃡}{G}}^{o}(\omega )$ elements for the host GaAs by incorporating the RIM phonons fitted to the INS data. For isolated defects of $T_{d}$ symmetry, the construction of full-size ${\overset{⃡}{G}}^{o}(\omega )$ and $\overset{⃡}{P}(\omega )$ matrices is achieved by decomposing them into blocks corresponding to the irreducible representations [57]:

(17) $${\Gamma_{T}}_{d}=\;A_{1}⊗\;E\;⊗\;F_{1}⊗\;3F_{2}.$$

In different irreducible representations, the impurity vibrational mode frequencies (e.g., in-band gap, local vibrational mode) are obtained by solving the real part of the determinantal equation [57]:

(18) $$\prod_{\mu \Gamma }det|[\overset{⃡}{I}-{\overset{⃡}{G}}_{\mu \Gamma }^{o}\left(\omega \right)\;{\overset{⃡}{P}}_{\mu \Gamma }\left(\omega \right)]|=0,$$

where the terms ${\overset{⃡}{G}}_{\mu \Gamma }^{o}$ (ω) and ${\overset{⃡}{P}}_{\mu \Gamma }\left(\omega \right)$ represent the ${\overset{⃡}{G}}^{o}(\omega )$ and $\overset{⃡}{P}(\omega )$ matrices projected onto the defect space in each of the irreducible ($A_{1}$, E, $F_{1}$, and $F_{2}$) representations. One must note that while the impurity modes in $A_{1}$, E, and $F_{2}$ representations are Raman active, the triply degenerate $F_{2}$ mode is, however, both IR and Raman active [69,70,71]. Using appropriate values of u (cf. Section 4.2.1 and Section 4.2.2) in GaAs, we calculated the LVMs of the triply degenerate $F_{2}$ mode for different light impurities occupying the As-sites. The RIM results compare reasonably well with the experimental data for isoelectronic ^14^N_As_ (~471 cm^−1^) and acceptor ^12^C_As_ (~582 cm^−1^) impurities [52].

Again, the values of LVMs in GaAs for N [69,70,71] and C-isotopic [72] defects have provided critical information about local atomic environments. Replacing the lighter ^14^N_As_ (^12^C_As_) isotopic masses with the heavier ^15^N_As_ (^13^C_As_) masses and retaining the same force constant changes u shifted the LVM to lower values, in excellent agreement with the IR reflectivity and Raman scattering results [69,70,71,72]. Based on a harmonic oscillator model, the frequency of an isotopic mass is inversely proportional to the square root of the mass of the vibrating atom. One can predict the local mode of a heavier isotopic mass from the lighter one using the ratio of their masses $\frac{\omega_{LVM}^{N^{14}}}{\omega_{LVM}^{N^{15}}}=\sqrt{\frac{M_{15}}{M_{14}}}\;.$ Given the masses of ^14^N and ^15^N (or ^12^C and ^13^C), the frequency of the heavier isotope ~452 cm^−1^ (~560 cm^−1^) is expected to be approximately 98.6% of the lighter isotopic mass frequency ~471 cm^−1^ (~582 cm^−1^). Our calculated result for the heavier ^15^N (^13^C) isotope ~458 cm^−1^ (~568 cm^−1^) agrees reasonably well with the above criteria and experimental data [69,70,71,72].

### 4.3. Reflectivity and Transmission Spectra

For GaAs_1-x_N_x_/GaAs, the TMM approach recently applied in [83] to study the R(ω) spectra in the FIR region of GS-MBE grown InAs_1-x-y_P_y_Sb_x_/n-InAs (001) epilayers is used here. The dielectric lattice response in materials without free charge carriers consists of simple sums of harmonic Lorentz oscillators. The amount of splitting between LO and TO modes is regarded as a measure of the polar strength of their respective phonon branches. In Section 3.2.1, the role of optical phonons in assessing the FIR dielectric response ${\tilde{\varepsilon }}_{l}\left(\omega \right)$ is described. The contribution of free carriers η to the dielectric function ${\tilde{\varepsilon }}_{e}\left(\omega \right)$ of film ${\tilde{\varepsilon }}_{f}\left(\omega \right)$ is written following the classical Drude approximation (see Equation (8)).

**Figure 4:**
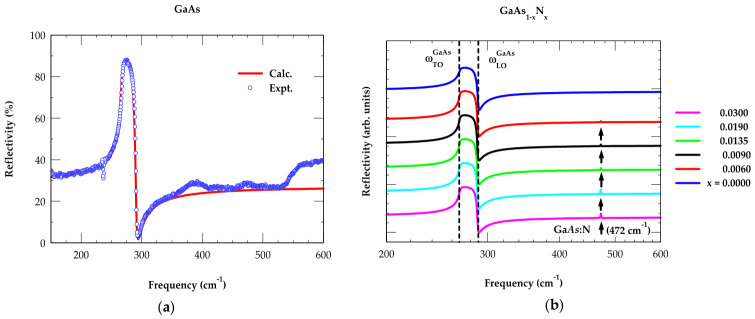
(**a**) Comparison of experimental (open blue color circles) and calculated (red line) R(ω) spectra of GaAs. (**b**) Composition-dependent R(ω) spectra of GaAs_1-x_N_x_/GaAs at near-normal incidence ($\theta_{i}=0$) showing a LVM of GaAs:N (TO_2_ of GaN sublattice) at 472 cm^−1^.

Equation (9) is used to evaluate ${\tilde{\varepsilon }}_{s}(\omega )$ and R(ω) for GaAs. The experimental R(ω) spectra (open blue circles) displayed in Figure 4a for the substrate compare reasonably well with the simulated (red line) results. Similar calculations for GaAs_1-x_N_x_/GaAs at near-normal incidence ($\theta_{i}=0$) are reported in Figure 4b for different N compositions x. The ω_LO1_ and ω_TO1_ phonon frequencies of GaAs are noticed at ~291.3 cm^−1^ and ~267.7 cm^−1^, respectively. The perusal of Figure 4b clearly shows the typical ω_LO1_ and ω_TO1_ phonons of the host GaAs lattice and the x-dependent TO_2_ mode of the GaN sublattice (vertical black arrows) presenting the LVM of GaAs:N near ~472 cm^−1^ [69,70,71].

**Figure 5:**
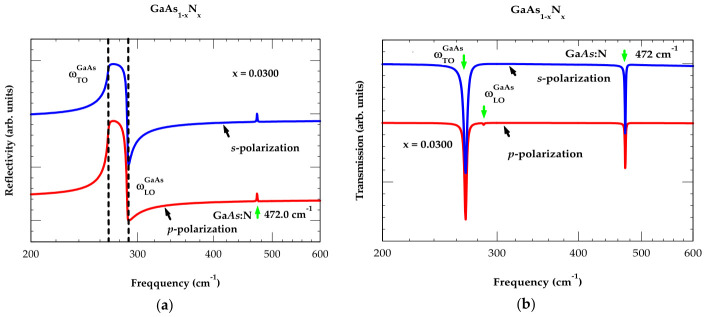
(**a**) Simulated results of s-polarized (blue line) and p-polarized (red line) R(ω) spectra for GaAs_1-x_N_x_/GaAs (x = 0.030) at oblique incidence $\theta_{i}=45{}^{\circ}$. (**b**) Simulated T(ω) results for GaAs_1-x_N_x_/GaAs at oblique incidence $\theta_{i}=45{}^{\circ}$ for s-polarized (blue line) and p-polarized (red color line) spectra, corroborating the Berreman effect [81].

In Figure 5a,b, we have reported the results of R(ω) and T(ω) spectra at oblique incidence ($\theta_{i}=45^{{}^{\circ}}$) for GaAs_1-x_N_x_/GaAs with x = 0.030. Comparison with Figure 4a,b offers a strong corroboration for the Berreman effect [81]. The *s*-polarization R(ω) (T(ω)) spectra unveil a sharp rise (minima) at ω_TO1_ of GaAs and ω_TO2_ of GaN, while in the p-polarization spectra, our simulation of T(ω) provided an additional dip at ω_LO1_ of GaAs (see Figure 5b). The study confirmed ω_LO1_ and ω_TO1_ phonons of GaAs and the x-dependent ω_TO2_ of the GaN sublattice (LVM of GaAs:N) at ~472 cm^−1^ [69,70,71].

### 4.4. Optical Constants in the UV Region

Optical constants in the UV region are required for semiconductor materials to design appropriate optoelectronic devices. Precise knowledge of the refractive indices [n$\left(E\right)$] and absorption coefficients [κ$\left(E\right)\;or\;\alpha$] for GaAs_1-x_N_x_ active layers is critical to attain the structures of LDs and PDs as these parameters strongly influence waveguide design, optical confinement, and overall device efficiency. In dilute GaAs_1-x_N_x_ we followed (cf. Section 3.3 and Section Modified Adachi’s Model Dielectric Function) a modified Adachi semi-empirical approach for studying its optical constants. While highly valued for its parameterization, the conventional Adachi method [43,44,45] suffers from several inherent physical and mathematical limitations, most notably (a) the use of Lorentzian broadening, (b) the omission of excitonic effects, and (c) the challenges with higher-energy critical points [41,42].

As described earlier (cf. Section 3.3 and Section Modified Adachi’s Model Dielectric Function), an extension to Adachi’s method suggested by Rakić and Majewski [46] is considered here. The incorporation of CPs with amplitudes $A_{k}$ of transition energies $E_{k},$ Gaussian broadening $\Gamma_{k}\;\left(E\right),$ and distribution parameters $\alpha_{k}$ has allowed us to accurately simulate x-dependent complex $\tilde{\varepsilon }$(E) of the ternary GaAs_1-x_N_x_ alloys. In the modified semi-empirical approach, the MDFs are empirically obtained using non-linear regression analysis (such as the Levenberg–Marquardt algorithm [75]) by fitting the calculated dielectric functions to the experimental SE data [42].

**Figure 6:**
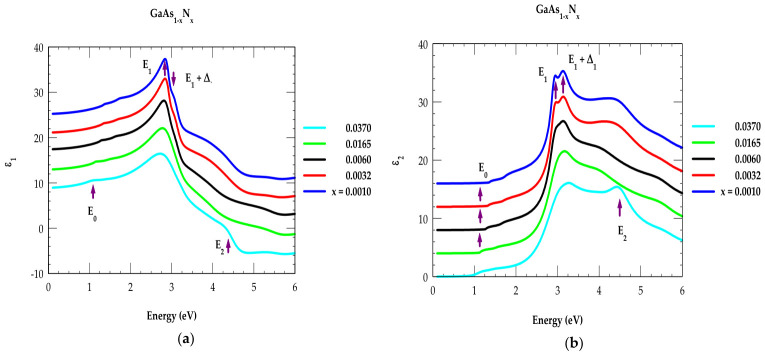

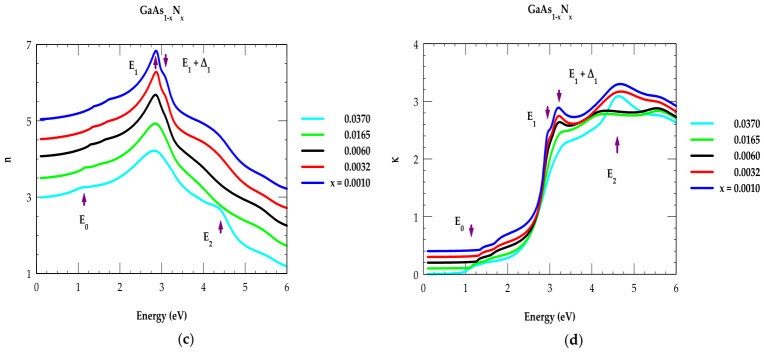
Based on Adachi’s modified MDFs, we have reported our simulated results for optical constants of dilute GaAs_1-x_N_x_ with different x: (**a**) $\varepsilon_{1}\left(E\right)$, (**b**) $\varepsilon_{2}\left(E\right)$, (**c**) n$\left(E\right)$, and (**d**) κ$\left(E\right)$ (see text).

Using this model, we have displayed the calculated results in Figure 6a,b for the real $\varepsilon_{1}\left(E\right)$ and imaginary $\varepsilon_{2}\left(E\right)$ parts of $\tilde{\varepsilon }$(E), respectively. Similar calculations for n$\left(E\right)$ and κ$\left(E\right)$ of $\tilde{n}$(E) are reported in Figure 6c,d. In Figure 6a–d, theoretical results are shifted upward for better legibility. Figure 6a characterizes the refractive behavior of $\varepsilon_{1}\left(E\right),$ showing typical peaks near $E_{0}$, and $E_{1}$ energies, while Figure 6b represents the absorptive features with crests reflecting the joint density of states at CPs. In Figure 6c,d, we have reported results for the refractive index (n) and absorption index (κ). A perusal of Figure 6a,b has revealed that $E_{1}$, and $E_{1}+\Delta_{1}$ CPs present dominating features at about ~3 eV, whereas the rising $\varepsilon_{2}$ values at 4.5 eV are caused by the CPs $E_{0}^{\prime }$ and $E_{2}$.

Except for a small deviation, the calculated spectral lines agreed very well with the SE spectra [39]. Simulated results for GaAs_1-x_N_x_ have accurately predicted (see Figure 6c,d) a redshift and accumulative broadening of the absorption edge as the N composition x increases, aligning with experimental observations [34]. The increased broadening of $E_{1}$ and $E_{1}+\Delta_{1}$ transitions can be attributed to the impurity-induced scattering of one-particle states and/or to virtual intermediate states [84,85,86,87].

#### Reflectance of GaAs_1-x_N_x_/GaAs Epifilms

For characterizing GaAs_1-x_N_x_/GaAs epifilms, measurements of optical reflectance R(E) in the UV region are a crucial, non-destructive and high-sensitivity tool, particularly for evaluating their bandgap energy, strain status, and optical quality. These valuable features are required for designing long-wavelength optoelectronic devices.

In the absence of experimental data, the calculation of reflectance spectra can be achieved by TMM [47] for a film of thickness $d_{f}$ considering the light interference effects using a three-layer system (i.e., air 1, a parallel film 2, and a substrate 3). The method requires Fresnel reflection coefficients between interfaces of “air-film” $r_{12},$ and “film-substrate” $r_{23}$ as well as appropriate values of the complex refractive indices ${\tilde{n}}_{i}\left(E\right)\left(with\;i=1\;to\;3\right).$ Under the condition of normal incidence $\theta_{i}=0$, the complex reflectivity coefficient (r) is used [47] for simulating $R\left(E\right)[\equiv \left|r^{2}\right|\equiv r.r^{*}]$.

**Figure 7:**
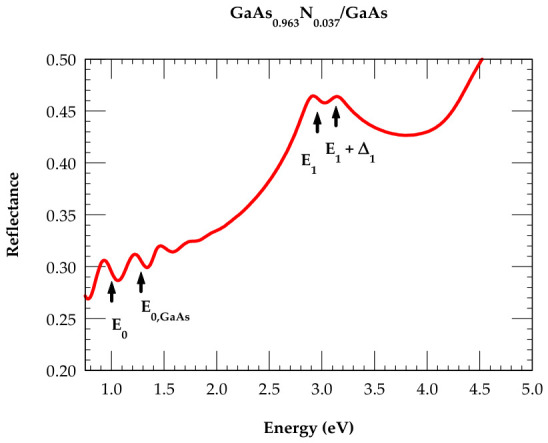
Using Adachi’s modified MDFs in TMM, we have reported reflectance R(E) spectra in the UV region for a 300 nm thick GaAs_0.963_N_0.037_ film overlaid on a GaAs substrate. The features noticed near $E_{0}\sim 1.0\;eV$ and $E_{0,GaAs}\;at\;$~1.45 eV originate from the bandgaps of the film and substrate, respectively. Dominating structures of $E_{1}$, and $E_{1}+\Delta_{1}$ CPs between 3 eV to 3.5 eV are in very good agreement with the optical constants of GaAs_1-x_N_x_ alloys (see text).

In our simulations, we have chosen ${\tilde{n}}_{1}\approx 1$ for air 1; for GaAs_1-x_N_x_ alloy film, the ${\tilde{n}}_{2}$ of layer 2 (see Section 4.4) depends upon the N concentration x and thickness $d_{f}$, while ${\tilde{n}}_{3}$ of layer 3 for the GaAs substrate has been achieved earlier with great accuracy [47]. Theoretical results of energy-dependent R(E) spectra for a 300 nm thick GaAs_0.963_N_0.037_ film (for instance) overlaid on a GaAs substrate are reported in Figure 7 (red line).

In the low-energy region, the features $E_{0}\sim 1.0\;eV$ and $E_{0,GaAs}\;at\;$~1.45 eV originate from the bandgaps of the film and substrate, respectively. The dominating structures of $E_{1}$, and $E_{1}+\Delta_{1}$ CPs between 3 eV to 3.5 eV are in very good agreement with the optical constants of GaAs_1-x_N_x_ alloys (see Figure 6a,d).

## 5. Concluding Remarks

Studying the basic characteristics of epitaxially grown III-V-N heterostructures [1,2,3,4,5,6,7,8,9,10] has been notoriously difficult due to phase separation, large miscibility gaps, and a high density of point defects, including nitrogen vacancies. These structural complexities significantly complicate the isolation of fundamental electrical and optical properties. Substituting a small amount of N onto the As sublattice in GaAs creates GaAs_1-x_N_x_, leading to a massive reduction in E_g_, which provides a powerful tool for band structure engineering. Highly mismatched dilute GaAs_1-x_N_x_ (x < 0.05) alloys are crucial for optoelectronics. Tensile strain combined with a strong N-induced conduction band anti-crossing effect allows for precise tuning of the optical properties. This synergistic approach has provided a resilient platform for developing long-wavelength LDs, PDs and high-efficiency MJ-SCs [1,2,3,4,5,6,7,8,9,10]. Epitaxial growth of GaAs_1-x_N_x_ on GaAs or Si substrates is a pivotal area of research for overcoming the limitations of conventional semiconductor materials, particularly for monolithic integration with mature Si complementary metal-oxide semiconductor (CMOS) technology. For telecommunication and high-efficiency tandem solar cells, the optimum performance of III-V-N based devices is heavily dependent on precisely managing their structural, lattice dynamical and optical characteristics. Optical bandgap, structural quality, surface morphology, and layer thickness d of GaAs_1-x_N_x_/GaAs epifilms are commonly influenced by x, interfacial strain, optical transitions and epitaxial growth conditions [11,12,13,14,15,16,17,18,19,20]. In nanostructured GaAsN films, the interaction of phonons with intrinsic and/or extrinsic charge carriers has been and still is a critical factor for evaluating their role in thermal management, thermoelectric energy conversion, and thermal insulation of various electronic devices.

Limited SE measurements on dilute GaAs_1-x_N_x_ alloys are the motive for us to methodically study $\omega_{j}\left(\vec{q}\right)$, R(ω), and T(ω) spectra in the FIR (100 cm^−1^–600 cm^−1^) range as well as evaluate optical constants in the UV (≤6 eV) region. High-resolution FTIR reflectivity measurements of GaAs are performed using a Bruker IFS 120 v/S FTIR spectrometer. A classical Lorentz–Drude model is justified in TMM for assessing the dielectric functions $\tilde{\varepsilon }\left(\omega \right)$ of GaAs_1-x_N_x_ epifilms and GaAs substrate. For GaAs_1-x_N_x_/GaAs (001) wafers (x ≤ 3.0%) this approach has enabled simulating R(ω) and T(ω) spectra in the FIR region at both the near-normal-($\theta_{i}=0)$ and oblique $(\theta_{i}\neq 0$) incidence. For low x, the spectra reveal typical ω_LO1_ and ω_TO1_ phonons of the GaAs substrate alongside a distinct x-dependent N-related ω_TO2_ mode near ~ 472 cm^−1^ as a LVM of N_As_ in GaAs:N. Moreover, in the GF framework, a realistic RIM has provided accurate N-isotopic shifts of LVMs, corroborating the FTIR results of $N_{As}$ in GaAs.

In the UV region, by adopting a semiempirical approach, we evaluated $\tilde{\varepsilon }\left(E\right)$, $\tilde{n}\left(E\right)\;of$ GaAs_1-x_N_x_ alloys and R(E) spectra of GaAs_1-x_N_x_/GaAs epilayers [40,41,42,43,44,45,46]. Simulated results for GaAs_1-x_N_x_ have accurately predicted the redshift and accumulative broadening of the absorption edge as N composition x increases, aligning with experimental observations [34]. The results of $\varepsilon_{1}\left(E\right),\;\;and\varepsilon_{2}\left(E\right)$ exhibited refractive and absorptive features showing typically the peaks near $E_{0}$ and $E_{1}$ energies as crests, reflecting the joint density of states at CPs in the BZ. Similar calculations of n(E) and κ(E) revealed that $E_{1}$ and $E_{1}+\Delta_{1}$ CPs present dominating features at about ~3 eV and the rising $\varepsilon_{2}$ values at 4.5 eV are caused by the CPs $E_{0}^{\prime }$ and $E_{2}$, in good agreement with SE measurements. The increase of broadening of $E_{1}$ and $E_{1}+\Delta_{1}$ transitions can be attributed to the impurity-induced scattering of one-particle states and/or to the virtual intermediate states [84,85,86,87].

## Figures and Tables

**Table 1:** Best-fit interatomic force constants of the rigid-ion model obtained using a methodology outlined in [49]. The IFCs are used for simulating the phonon dispersions $\omega_{j}\left(\vec{q}\right)\;$ and one-phonon density of states for the zb GaAs and GaN materials.

Parameter ^(*a*)^	GaAs	GaN
A	−0.4071	−0.6648
B	−0.166	−0.505
C_1_	−0.0177	−0.0600
C_2_	−0.0461	−0.1004
D_1_	0.0248	0.0162
D_2_	−0.1233	−0.1880
E_1_	0.0912	0.0900
E_2_	0.0834	0.135
F_1_	−0.1172	−0.0460
F_2_	0.2008	0.185
Z_eff_	0.658	1.15

^(*a*)^ Refs. [48,49].

**Table 2:** Basic characteristics of zb GaAs and GaN including lattice constant ($a_{0}$) in Å; elastic constants ($c_{ij}$); and bulk modulus (B) in 10^11^ dyn/cm^2^. Inelastic neutron scattering (INS) phonon frequencies (in cm^−1^) at high-symmetry points Γ, X, and L are listed. Phonon modes calculated by RIM are compared with INS and other theoretical data.

GaAs				GaN		
Parameter	INS ^(*a*)^	RIM ^(*b*)^	Others ^(*c*)^	Ab-Initio ^(*d*)^	RIM ^(*b*)^	Others ^(*c*)^
$a_{0}$	5.6531	5.65	5.65	4.447	4.5	4.413–4.518
B	7.69	7.64	7.8–9.73	19.6	19.1	19.0–20.2
$c_{11}$	12.11	11.97	10.2–13.94	28.1	27.6	26.4–29.7
$c_{12}$	5.48	5.47	3.60–7.62	15.3	14.9	12.6–15.4
$c_{44}$	6.04	6.10	4.70–6.64	167	15.9	15.8–20.6
$\omega_{LO(\Gamma )}$	293.0	292.0	287–293	750	743	740–752
$\omega_{TO(\Gamma )}$	271.0	268.7	260–270	560	552	540–560
$\omega_{LO(X)}$	240.0	236.2	231–238		714	710–715
$\omega_{TO(X)}$	256.3	257.8	250–260		626.4	620–630
$\omega_{LA(X)}$	225.0	221.2	218–223		343.6	340–347
$\omega_{TA(X)}$	81.7	77.8	76–80		195.5	187–197
$\omega_{LO(L)}$	241.7	253.2	230–252	720	718	715–720
$\omega_{TO(L)}$	263.3	256.1	245–260	585	607	585–610
$\omega_{LA(L)}$	206.7	206.1	196–205	345	340	340–347
$\omega_{TA(L)}$	63.3	59.4	55–65	139	137	135–140

^(*a*)^ Ref. [78] ^(*b*)^ Our ^(*c*)^ Refs. [77,78] ^(*d*)^ Ref. [76].

**Table 3:** The best-fit Lorentz–Drude parameter values used to simulate the x-dependent R(ω) and T(ω) spectra for dilute GaAs_1-x_N_x_ ternary alloys and GaAs substrate (see text).

GaAs_1-x_N_x_						
Parameter	GaAs	x = 0.006	x = 0.009	x = 0.0135	x = 0.019	x = 0.030
$\varepsilon_{\infty }$	12.6	12.7	12.8	12.6	12.4	12.3
$\omega_{TO1}$	267.7	268	268	268	268	268
$\omega_{LO1}$	291.3	293	292	292	291	292
Γ_1_	3.7	4	3.8	2.7	3.9	4.1
$\omega_{TO2}$		472	472	472	472	472
Γ_2_		2.6	2.5	2.4	2.6	2.5

## Data Availability

The original contributions presented in this study are included in the article. Further inquiries can be directed to the corresponding author.
